# P-1268. Evaluation of Mpox Coinfections with Hepatitis B, Hepatitis C, HIV, and Sexually Transmitted Infections During the Outbreak Peak In New York City

**DOI:** 10.1093/ofid/ofae631.1449

**Published:** 2025-01-29

**Authors:** Alex J Pelliccione, Kimberly Johnson, Tristan D McPherson, Preeti Pathela, Sarah Braunstein

**Affiliations:** NYU Grossman School of Medicine, New York, New York; New York City Department of Mental Health and Hygiene, New York City, New York; New York City Department of Health and Mental Hygiene, New York, NY; New York City Department of Health and Mental Hygiene, New York, NY; New York City Department of Health and Mental Hygiene, New York, NY

## Abstract

**Background:**

New York City (NYC) was an epicenter of the 2022 global mpox outbreak. Mpox is spread through skin-to-skin contact, including sexual contact, and there is a high prevalence of coinfections with HIV and other sexually transmitted infections (STI). The frequency of mpox and Hepatitis B/C virus (HBV/HCV) coinfections is unknown, though HBV/HCV can be sexually transmitted, and HBV/HCV prevention and treatment are part of syndemic approaches with HIV/STI. We evaluated mpox coinfections in NYC, focusing on HBV/HCV.

**Figure:**
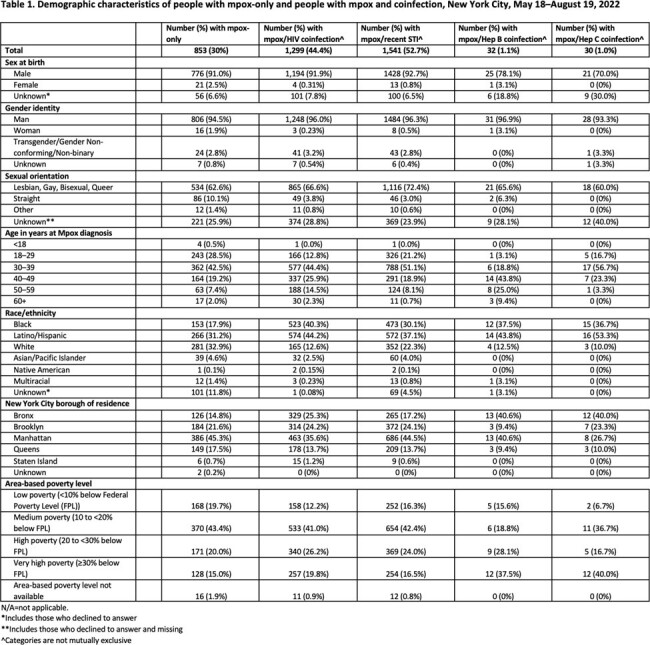

Demographic characteristics of people with mpox-only and people with mpox and coinfection, New York City, May 18–August 19, 2022

**Methods:**

We performed descriptive analyses and assessed coinfections using matched NYC Health Department surveillance registries for HBV/HCV, HIV, STI (chlamydia, gonorrhea, early syphilis), and mpox during the outbreak peak (May 18–August 19, 2022). Registries included all patient laboratory reports for each infection and demographics: age at mpox diagnosis, sex at birth, gender identity, race/ethnicity, NYC borough of residence, and area-based poverty level. People with resolved HCV were excluded.

**Results:**

During the analytic period, 2925 people were diagnosed with mpox; 1299 (44.4%) were living with diagnosed HIV, 32 (1.1%) with HBV, and 30 (1.0%) with HCV, with half (1541; 52.7%) diagnosed with STI in the prior 12 months (Table 1). Coinfections of mpox and HBV/HCV were more common for people living in very high poverty areas (HBV-mpox: 37.5%, HCV-mpox: 40%). For non-Hispanic Black and Latino/Hispanic people, mpox coinfections with HIV (40.3% and 44.2%, respectively); HBV (37.5%; 43.8%); HCV (36.7%; 53.3%); and recent STI (30.1%, 37.1%) were more frequent than mpox monoinfection (17.9%; 31.2%). For non-Hispanic white people, coinfections with HIV (12.6%); HBV (12.5%); HCV (10%); and recent STI (22.3%) were less frequent than mpox monoinfection (32.9%).

**Conclusion:**

Among people diagnosed with mpox, coinfection rates with HBV/HCV were low compared with HIV/STI. This might reflect sexual transmission as a less common driver of HBV/HCV infection in communities affected by mpox. There were more coinfections in Black and Latino/Hispanic people and more HBV/HCV coinfections in people living in very high poverty areas. Place-based HBV/HCV screening could be beneficial in future mpox outbreaks, and universal HIV/STI screening at mpox diagnosis should continue.

**Disclosures:**

**All Authors**: No reported disclosures

